# Case Report: Epididymal NK/T-cell lymphoma and adrenal diffuse large B-cell lymphoma are misdiagnosed as tuberculosis: two case reports and literature review

**DOI:** 10.3389/fonc.2025.1529049

**Published:** 2025-06-03

**Authors:** Dan Ye, Xuhui Liu, Yuexiang Yang, Yang Yang, Zhentao Fei, Huarui Liu, Qilin Zhan, Lu Xia

**Affiliations:** ^1^ Department of Tuberculosis, Shanghai Public Health Clinical Center, Shanghai, China; ^2^ Department of Pathology, Shanghai Public Health Clinical Center, Shanghai, China; ^3^ Department of Hematology, Shanghai Public Health Clinical Center, Shanghai, China

**Keywords:** epididymal lymphoma, adrenal lymphoma, epididymal tuberculosis, adrenal tuberculosis, misdiagnosis

## Abstract

Extranodal NK/T-cell lymphoma(ENKTCL) and diffuse large B-cell lymphoma(DLBCL) are specific subtypes of non-Hodgkin lymphoma(NHL), which lack specific features and are difficult to diagnose. The clinical features of lymphoma and tuberculosis are similar, which are easy to be misdiagnosed and lead to delayed treatment. This report describes two cases, one that of a 34-year-old man who was diagnosed with epididymal tuberculosis because of fever, progressive epididymal enlargement, positive T-cell Spot Test(T-SPOT), and epididymal magnetic resonance imaging(MRI) suggesting possible epididymal tuberculosis. He was treated with anti-tuberculosis therapy for 1 month, but the patient’s epididymis continued to grow. Needle biopsy pathology and immunochemical examination showed an epididymal NK/T cell lymphoma, which gradually shrank after chemotherapy. Meanwhile, a 77-year-old female patient was reported who was diagnosed with adrenal tuberculosis because of fever, night sweats, abdominal pain, positive QuantiFERON-TB Gold(QFT) test, and adrenal tuberculosis detected by positron emission tomography/computed tomography(PET/CT). She received anti-tuberculosis treatment for 2 weeks, but her symptoms were not improved. Biopsy pathology and immunochemical examination showed adrenal diffuse large B-cell lymphoma, which deteriorated rapidly after chemotherapy and she finally died. In this report, epididymal NK/T cell lymphoma and adrenal diffuse large B-cell lymphoma are rare, and the disease develops rapidly. The diagnosis depends on pathological morphology and immunohistochemistry. Early detection, diagnosis, and treatment are crucial for the prognosis of patients.

## 1 Introduction

Lymphoma is a group of diseases with malignant proliferation of lymphocytes, which are mainly divided into non-Hodgkin lymphoma (NHL) and Hodgkin lymphoma (HL) (1). Extranodal natural killer/T-cell lymphoma (ENKTCL) is a subtype of NHL. It often involves the human nasal cavity but can also be rarely seen in the human lung, adrenal gland, testis, and other body parts (2). Primary testicular lymphoma accounts for 1%-2% of NHL, 80% of which are diffuse large B-cell lymphoma (DLBCL). Testicular NK/T cell lymphoma is rare, and epididymal NK/T cell lymphoma is even rarer (3). DLBCL is a common subtype of NHL, with an incidence of about 25-40% (4). Patients usually present with progressively enlarged lymph nodes or extranodal space-occupying lesions. About 60% of patients have primary tumors in lymph nodes and 40% in extranodal organs. About 25% of DLBCL are found to involve the adrenal glands on autopsy, but primary adrenal lymphomas are rare, accounting for only 1% (5). Tuberculosis (TB) is a specific infectious disease caused by Mycobacterium tuberculosis, which can occur in all body systems, most commonly in the lungs (6). Extrapulmonary tuberculosis(EPTB) accounts for only 15% (7), and epididymal and adrenal tuberculosis belong to EPTB. The clinical features of lymphoma and TB are similar, such as fever, night sweats, fatigue, and unexplained weight loss. When combined with tuberculosis infection, pathological biopsy is not easy to obtain; lymphoma is easily misdiagnosed as TB, leading to delayed treatment and affecting the prognosis of patients (8). This report discusses the clinical presentation and diagnosis of epididymal NK/T-cell lymphoma and diffuse large B-cell lymphoma of the adrenal gland, as well as a review of the literature on the misdiagnosis of lymphoma and tuberculosis to minimize leakage and misdiagnosis to assist in proper clinical treatment, and to improve patient survival.

## 2 Case presentation

### 2.1 Case 1

**Figure 1:**
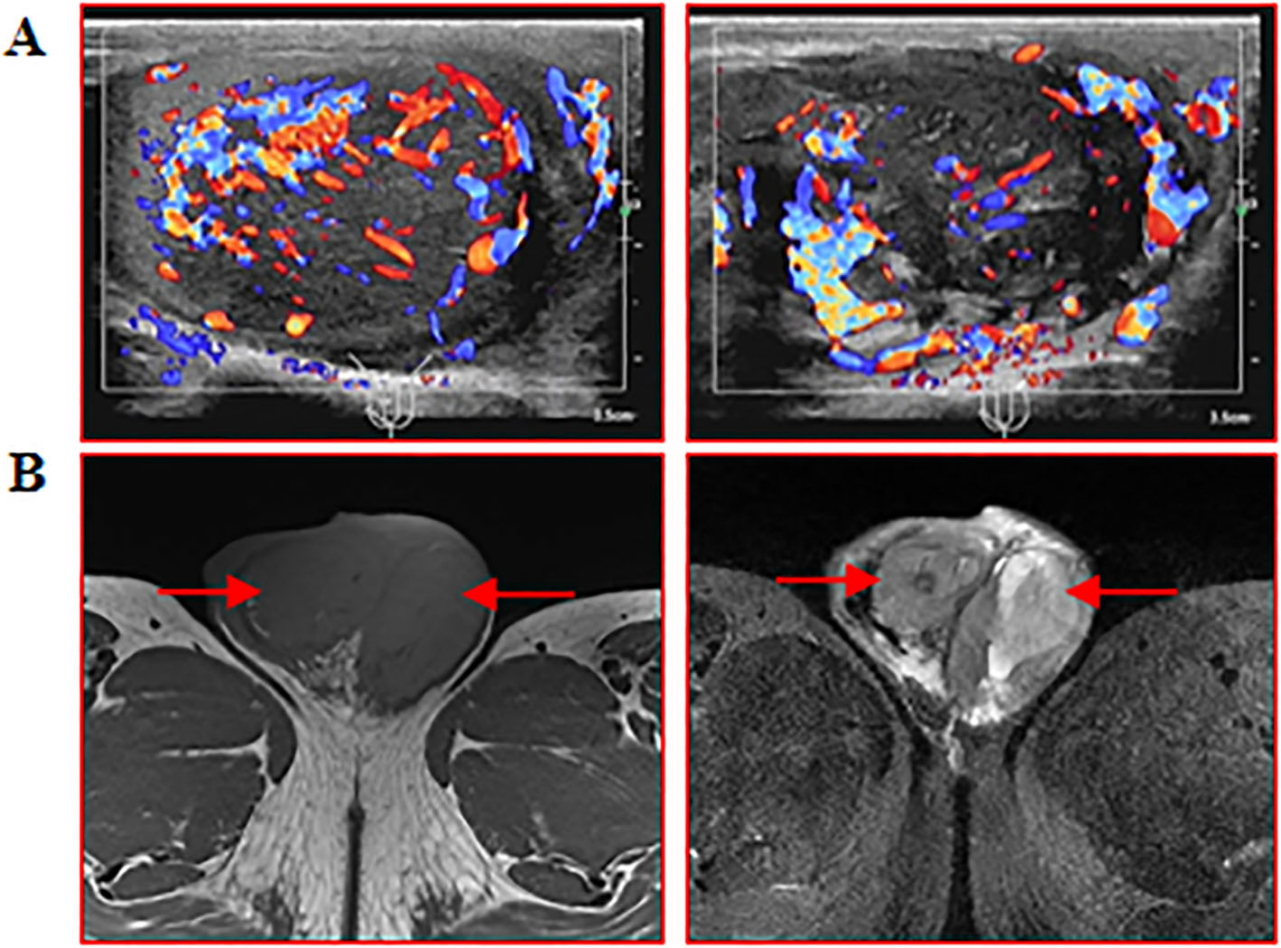
Ultrasonography and MRI of the testis and epididymis were performed. **(A)** Testis and epididymis B ultrasound: scale bar, 3.5 cm; **(B)** MRI of testis and epididymis: red arrows indicate enlarged testis and epididymis.

**Figure 2:**
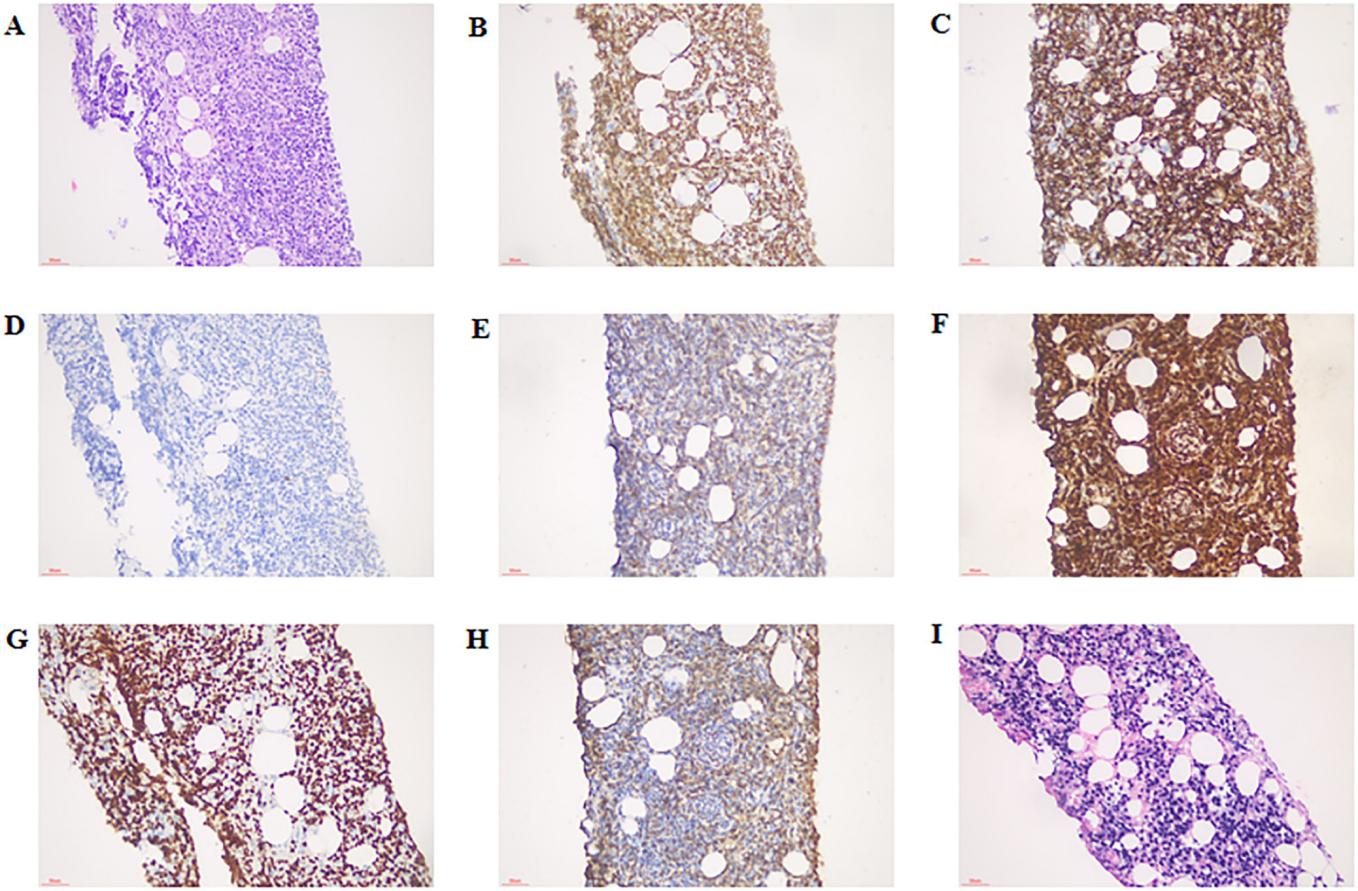
Immunostaining for cytokines in the right epididymis. **(A)** HE staining, × 200 magnification; **(B)** CD3 positive staining, ×200 magnification; **(C)** CD8 positive staining, ×200 magnification; **(D)** CD20 positive staining, ×200 magnification; **(E)** CD56 positive staining, ×200 magnification; **(F)** GranB positive staining ×200 magnification; **(G)** Ki-67 positive staining, ×200 magnification; **(H)** TIA-1 positive staining, ×200 magnification; **(I)** EBER (molecular diagnostics) positive staining, ×200 magnification. HE, hematoxylin-eosin.

A 34-year-old male patient was admitted to another hospital for fever and gradual enlargement of epididymis in August. The patient’s T-cell Spot Test(T-SPOT) and Purified Protein Derivative(PPD) tests were positive. Ultrasonography of the testis and epididymis showed irregular enlargement of the right and epididymis; a hypoechoic area in the right testis was 41×28mm, and the tail of the right epididymis was 28×27mm (**Figure 1A**). Magnetic Resonance Imaging(MRI) of the testis and epididymis showed soft tissue shadows in both testes, which showed equal hypointensity on T1WI equal hypointensity and uneven signal intensity on fat-suppression T2, and slightly higher signal intensity on DWI (**Figure 1B**). The possibility of epididymal tuberculosis was considered in other hospitals. The patient was treated with anti-tuberculosis therapy, and the epididymis continued to enlarge after 1 month of treatment. He subsequently visited our hospital; the laboratory test results are shown in **Table 1**. The pathology of the right epididymis puncture biopsy suggested atypical hyperplasia of T lymphocytes. The immunohistochemistry findings demonstrate positivity for CD3, CD20, Ki-67 (80%), CK, CD68, CD8, CD4, CD5, CD79a, CD56, GranB, TIA-1, BCL-2, and BCL-6, while WT-1 and CD10 are negative. Molecular pathology confirms positivity for EBER (**Figure 2**). Combined with immunohistochemistry and *in situ* hybridization results, ependymal NK/T-cell lymphoma diagnosis was made. The patient received chemotherapy and radiotherapy for 3 months, and the epididymis gradually shrunk.

**Table 1:** Laboratory results of epididymal NK/T-cell lymphoma patients' findings on admission.

Laboratory indicator	Normal range	Result
WBC, x10^9^/L	3.50-9.50	5.06
Hb, g/L	130.00-175.00	133.00
PLT, x10^9^/L	125.00-350.00	253.00
ALT, U/L	9.00-50.00	63.10
AST, U/L	15.00-40.00	27.20
TBIL, umol/L	≤23.00	6.50
DBIL, umol/L	≤8.00	3.10
Albumin, g/L	40.00-55.00	46.00
Potassium, mmol/L	3.50-5.30	4.50
Sodium, mmol/L	137.00-147.00	137.90
Chloride, mmol/L	99.00-110.00	103.30
Creatinine, umol/L	57.00-97.00	71.00
Uric Acid, umol/L	208.00-428.00	357.00
eGFR, ml/(min*1.73m^2^)	>90.00	117.09
hs-CRP, mg/L	0.00-10.00	3.64
ESR, mm/h	0.00-15.00	35.00
CEA, ng/ml	≤5.20	0.90
AFP, ng/ml	≤7.00	5.32

WBC, White Blood Cell Count; Hb, Hemoglobin; PLT, Platelet Count; ALT, Alanine Aminotransferase; AST, Aspartate Aminotransferase; TBIL, Total Bilirubin; DBIL, Direct Bilirubin; eGFR, estimated Glomerular Filtration Rate; hs-CRP, High-sensitivity C-reactive Protein; ESR, Erythrocyte Sedimentation Rate; CEA, Carcinoembryonic Antigen; AFP, Alpha-fetoprotein.

### 2.2 Case 2

**Figure 3:**
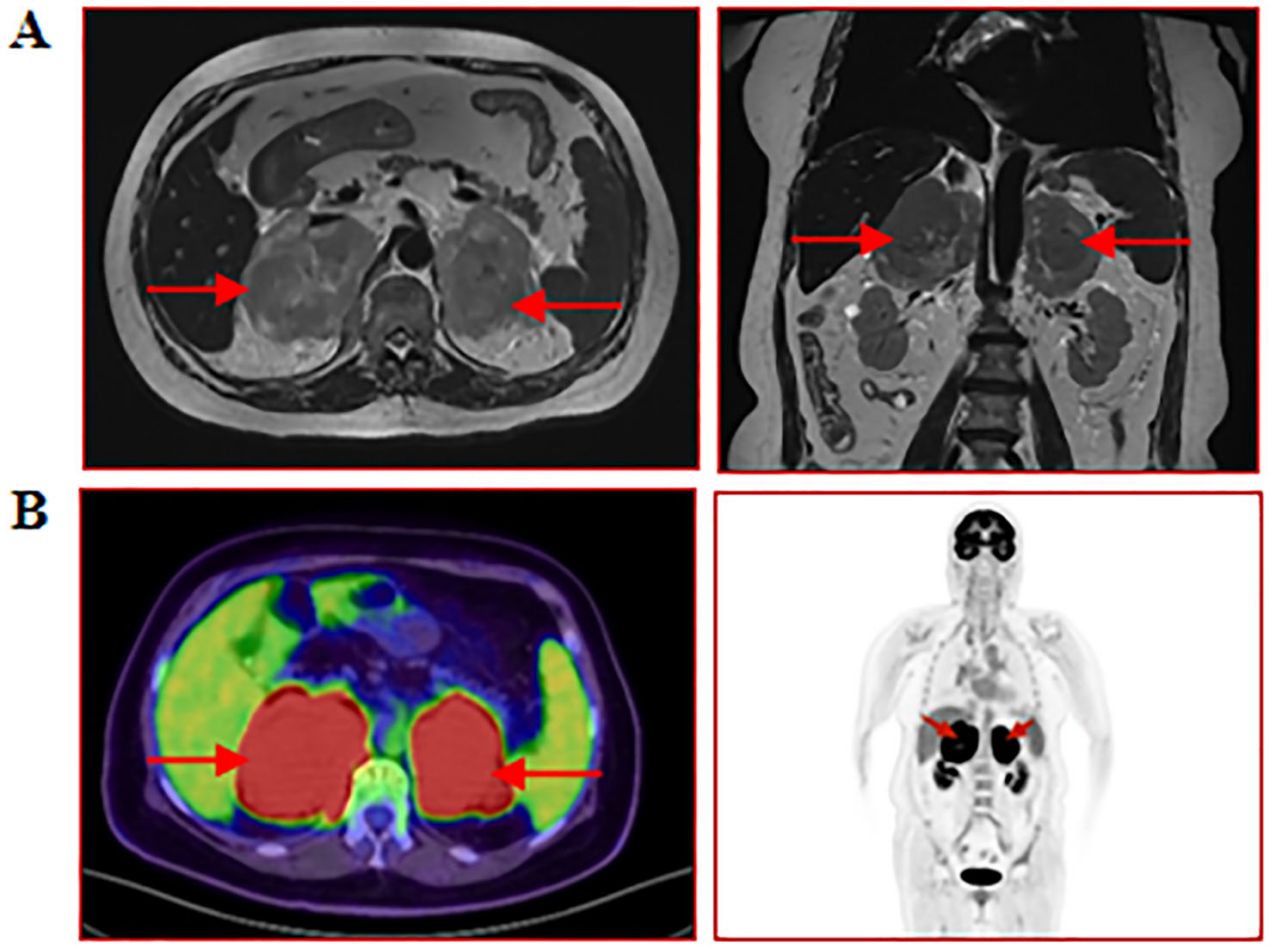
MRI and PET/CT of the adrenal glands. **(A)** Cross-sectional and coronal MRI images of the adrenal gland; **(B)** Coronal PET/CT images of the adrenal gland. The red arrow indicates the enlarged adrenal gland.

**Figure 4:**
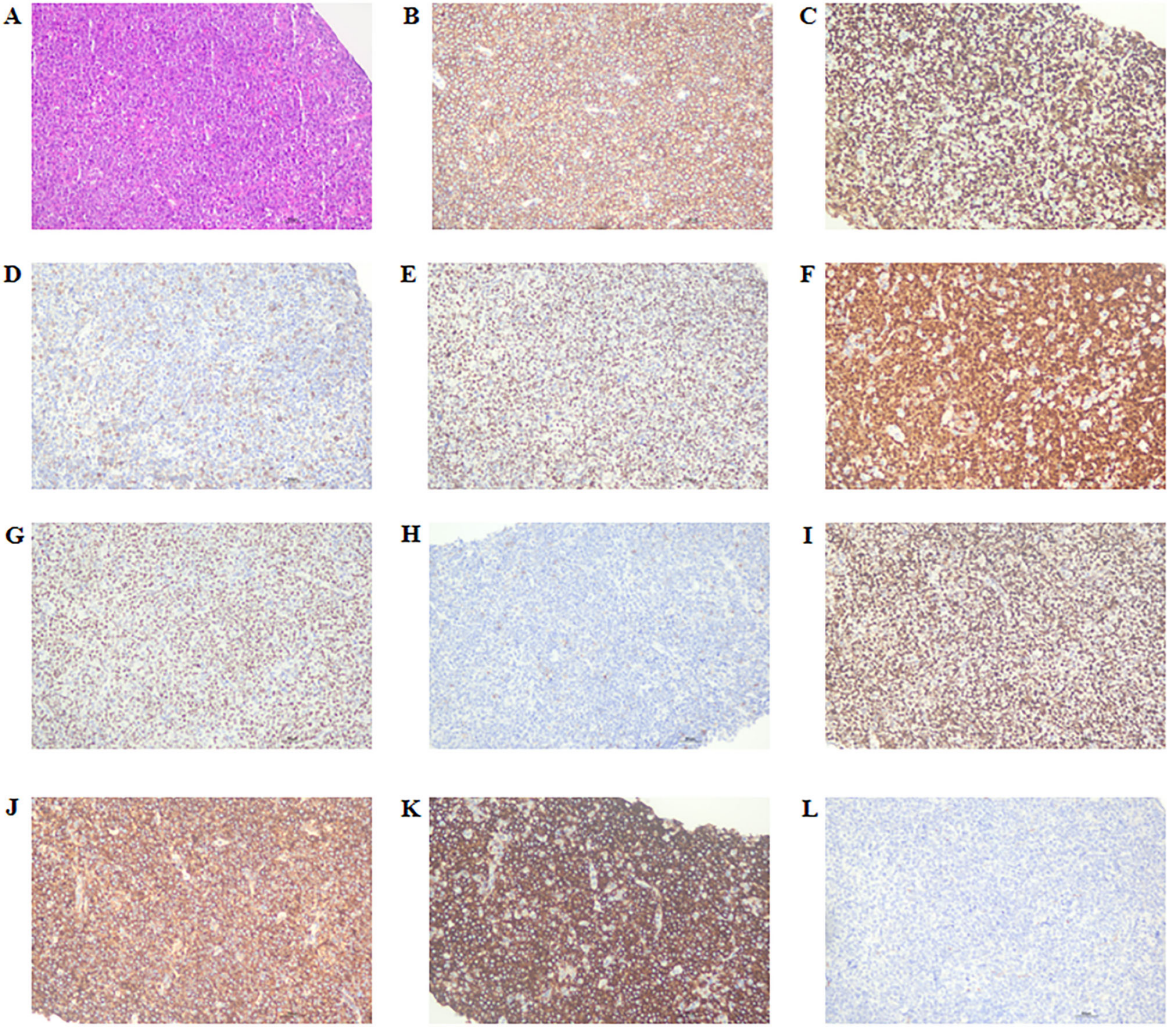
Immunostaining in the right adrenal gland for cytokines. **(A)** HE staining, × 200 magnification; **(B)** CD20 positive staining, ×200 magnification; **(C)** Ki-67 positive staining, ×200 magnification; **(D)** Bcl-2 positive staining, ×200 magnification; **(E)** Bcl-6 positive staining, ×200 magnification; **(F)** MUM-1 positive staining, ×200 magnification; **(G)** C-myc positive staining, ×200 magnification; **(H)** CD15 positive staining, ×200 magnification; **(I)** P53 positive staining, ×200 magnification; **(J)** CD22 positive staining, ×200 magnification; **(K)** CD19 positive staining, ×200 magnification; **(L)** CD10 negative staining, ×200 magnification. HE, hematoxylin-eosin.

A 77-year-old female patient was admitted to another hospital due to afternoon fever, night sweats, nausea, vomiting, poor appetite, abdominal distension, abdominal pain, dizziness, fatigue, listlessness, insomnia, and weight loss for 20 days. The patient’s QuantiFERON-TB Gold(QFT) test was positive. Adrenal MRI revealed that the left and right sizes were approximately 73×73×51mm and 79×87×63mm, respectively. T1WI showed a slow signal, T2W, fat-suppression T2W showed an isointense and slightly hyperintense mixed signal, and an enhanced scan showed no noticeable enhancement (**Figure 3A**). Positron emission tomography/computed tomography (PET/CT) showed a soft tissue density mass of 67×47mm and 83×52mm in the left and right adrenal regions, respectively, with a flocculent blur surrounding it, and increased FDG metabolism with a max value of 32.6 to 33.6 (**Figure 3B**). The patient was treated with anti-tuberculosis therapy. There was no significant improvement in symptoms after 2 weeks of treatment. He subsequently visited our hospital; the laboratory test results are shown in **Table 2**. A right adrenal gland puncture biopsy suggested antacid staining (-) and PAS staining (-). The immunohistochemistry results show positivity for CD20, CD79a, Ki-67 (90%), Bcl-2, Bcl-6, MUM-1, C-myc, CD15, P53, CD22, and CD19, while CD3, CD5, CD10, ALK, CD30, HHV8, CD38, CyclinD1, and EMA are negative (**Figure 4**). *In situ* hybridization: EBER(-).FISH: The BCL6 isolation probe was negative, with a total number of 100 cells, of which 1% were isolated cells; the BCL-2 isolation probe was negative, with a total number of 100 cells, of which 2% were isolated cells; and MYC isolation probe was positive, with a total number of 105 cells, of which 71.43% were isolated cells. Combined with morphology, immunohistochemistry, and fluorescence *in situ* hybridization, the diagnosis of adrenal diffuse large B-cell lymphoma, the non-GCB type, was made. The patient was treated with chemotherapy but unfortunately died due to rapid deterioration 81 days after the initial symptoms.

**Table 2:** Laboratory results of adrenal diffuse large B-cell lymphoma patients' findings on admission.

Laboratory indicator	Normal range	Result
WBC, x10^9^/L	3.50-9.50	4.75
Hb, g/L	115.00-150.00	77.00
PLT, x10^9^/L	125.00-350.00	96.00
ALT, U/L	7.00-40.00	11.60
AST, U/L	13.00-35.00	32.60
TBIL, umol/L	≤23.00	27.30
DBIL, umol/L	≤8.00	19.40
Albumin, g/L	40.00-55.00	29.60
Potassium, mmol/L	3.50-5.30	3.96
Sodium, mmol/L	137.00-147.00	121.60
Chloride, mmol/L	99.00-110.00	90.00
Creatinine, umol/L	41.00-81.00	84.00
Uric Acid, umol/L	155.00-357.00	336.00
eGFR, ml/(min*1.73m^2^)	>90.00	81.68
hs-CRP, mg/L	0.00-10.00	111.43
ESR, mm/h	0.00-20.00	47.00
CEA, ng/ml	≤5.20	0.47
AFP, ng/ml	≤7.00	1.32
Cortisol, ug/dl	4.26-24.85	3.51
ACTH, pg/ml	7.20-63.3	495.71

WBC, White Blood Cell Count; Hb, Hemoglobin; PLT, Platelet Count; ALT, Alanine Aminotransferase; AST, Aspartate Aminotransferase; TBIL, Total Bilirubin; DBIL, Direct Bilirubin; eGFR, estimated Glomerular Filtration Rate; hs-CRP, High-sensitivity C-reactive Protein; ESR, Erythrocyte Sedimentation Rate; CEA, Carcinoembryonic Antigen; AFP, Alpha-fetoprotein; ACTH, Adrenocorticotropic Hormone.

## 3 Discussion

ENKTCL can be divided into nasal type (involving the nasal cavity and upper respiratory tract), non-nasal type (involving the skin, gastrointestinal tract, testes, and other organs), and disseminated type (involving multiple organs) (9). The incidence of ENKTCL varies significantly among different ethnic groups, and the disease mainly occurs in Asia (especially Japan, China, and Korea) and Central and South America. It is rarer in other regions (10). The pathogenesis of ENKTCL is mainly related to Epstein-Barr virus (EBV) infection, related gene abnormalities, abnormal activation of signaling pathways, and changes in the tumor microenvironment. The clinical manifestations of ENKTCL are atypical, and it is not easy to diagnose and treat early. About 30%-40% of patients will be accompanied by systemic symptoms such as fever, night sweats, and weight loss (11). The pathological features of ENKTCL are coagulation necrosis centered on blood vessels, mixed infiltration of various inflammatory cells, and panischemic necrosis of normal tissues. The EBV detection rate of ENKTCL is as high as 90%, which is this pathological type’s most important clinical feature (12). The pathology-specific immunophenotypes of ENKTCL showed CD56(+), CD3ϵ(+), TIA-1(+), Granzyme B(+), perforin(+), Ki-67(+), and EBER hybridisation(+) (13, 14). ENKTCL is highly invasive and vascular destructive, highly aggressive, and has a poor prognosis. The 5-year overall survival rate of nasal-type patients is about 54%, while the 5-year overall survival rate of non-nasal-type patients is only 34% (15). Epididymal NK/T cell lymphoma is sporadic among non-nasal type extranodal NK/T cell lymphomas, with only one documented case involving the epididymis reported to date. DU W et al. described a case in which a cytological examination of a right scrotal mass puncture performed at another institution initially suggested right epididymal tuberculosis. The PPD test was negative. To achieve a definitive diagnosis, a right epididymectomy was performed, and postoperative pathological assessment identified the condition as right epididymal non-Hodgkin lymphoma, specifically NK/T-cell lymphoma (16). Epididymal tuberculosis usually has an insidious onset and a long course of disease and often manifests as a unilateral painless mass with or without urinary tract irritation symptoms. Epididymal tuberculosis is difficult to differentiate from epididymal lymphoma only by clinical manifestations and physical examination. Clinically, epididymal tuberculosis should be suspected when there is a history of tuberculosis infection, the T-SPOT test or QFT test result is positive, epididymal CT examination shows epididymal enlargement, ring lesions, and nodular enhancement, and general antibiotic treatment is poor. However, an epididymal tumor should be suspected if the mass rapidly grows, with ill-defined borders, firm texture on palpation, and irregular surface characteristics. However, the final diagnosis was based on pathological examination (17). In general, due to the lack of specific clinical manifestations, imaging examination, and complex pathology, epididymal NK/T cell lymphoma easily leads to misdiagnosis and missed diagnosis, which should be paid enough attention in the diagnosis and treatment. Accurate pathological examination is the main basis for diagnosis. Early detection and diagnosis are of great significance for improving the survival of patients.

Primary Adrenal lymphoma (PAL) is usually bilateral than unilateral patients, accounting for 75% of the total incidence of PAL. The male-to-female ratio is 2∶1, and the elderly are more commonly affected (18). The etiology of PAL is still unclear, and its occurrence may be related to hematopoietic system abnormalities, immune system disorders, viral infections, and gene mutations. PAL lacks specific symptoms and signs and often manifests as abdominal pain, abdominal distension, lower back discomfort, unexplained fever, poor appetite and fatigue, and weight loss (19). DLBCL is the most common subtype of PAL, accounting for about 75% of all PAL cases. Histologically, the adrenal tissue is replaced by diffuse large lymphocytes. Based on the expression of related proteins detected by IHC, Hans classification system divided DLBCL into the following Germinal Center B-cell-like(GCB) type: CD10(+) or CD10(-), BCL6 (+)/MUM-1(-), and non-Germinal Center B-cell like(non-GCB) type: CD10(-), BCL6 (+), MUM-1 (+) or CD10(-), BCL6 (+) (20), non-GCB type DLBCL is often considered to be associated with poor prognosis (21). Adrenal diffuse large B-cell lymphoma (non-GCB type) is a relatively rare lymphoma, and its clinical manifestations are similar to adrenal tuberculosis. Jiang LM et al. reported a case involving a patient with a history of tuberculosis exposure and a positive T-SPOT test. Adrenal CT revealed bilateral adrenal gland enlargement, while adrenal biopsy pathology suggested granulomatous inflammation. The patient was clinically diagnosed with adrenal tuberculosis; however, no improvement was observed following anti-tuberculosis therapy. Subsequent lymphoma-related immunohistochemical analysis of the original specimen confirmed a diagnosis of adrenal peripheral T-cell lymphoma with granulomatous features (22). Adrenal tuberculosis is usually transmitted to the adrenal gland by Mycobacterium tuberculosis through the blood. Up to 12% of patients with adrenal tuberculosis have an asymptomatic infection, and clinical signs associated with primary adrenal insufficiency become evident only when more than 90% of the adrenal glands have been destroyed. Common symptoms included fatigue, weight loss, nausea, vomiting, diarrhea, abdominal pain, hyponatremia, and hyperkalemia. In the early stage of adrenal destruction by Mycobacterium tuberculosis, mass-like enlargement may occur, which should be differentiated from adrenal lymphoma. When the patient has evidence of tuberculosis infection, the T-SPOT test or QFT test results are positive, and the bilateral adrenal CT scan shows signs of nodular enlargement, thickening, and calcification, adrenal tuberculosis should be suspected, but the final diagnosis is based on pathological examination. Adrenal diffuse large B-cell lymphoma is a kind of malignant tumor with rapid progress and poor prognosis. Due to the atypical clinical manifestations and imaging examination, the incidence is low, which can easily be missed or misdiagnosed. Therefore, for patients with symptoms such as abdominal pain, unexplained fever, and weight loss, when unilateral or bilateral adrenal masses are found, this disease should be considered, and needle biopsy or surgery should be performed in time to confirm the diagnosis.

We reviewed PubMed and Web of Science papers using “lymphoma” AND “tuberculosis.” Only Case Reports were included. Based on the search results, **Table 3** chronologically summarizes cases of misdiagnosis between lymphoma and tuberculosis reported in the English literature. Seventeen cases were identified, with disease sites including the prostate, central nervous system, lungs, lymph nodes, larynx, abdomen, stomach, and vertebrae (23–39). Among these, nine patients with lymphoma were initially misdiagnosed with tuberculosis. Four of these patients succumbed to the disease after receiving only anti-tuberculosis therapy, having been clinically diagnosed with tuberculosis based on medical history, physical examination, and auxiliary tests. Their conditions deteriorated following diagnostic anti-tuberculosis treatment and final histopathological and immunohistochemical analyses confirmed lymphoma. The remaining five lymphoma patients showed clinical improvement following appropriate management, including chemotherapy, bone marrow transplantation, or surgical intervention upon definitive lymphoma diagnosis. Conversely, eight cases of tuberculosis were initially misdiagnosed as lymphoma, but correct diagnosis and subsequent anti-tuberculosis treatment resulted in significant clinical improvement. Min Bai et al. described a patient whose initial biopsy pathology suggested prostate lymphoma. Following R-CHOP chemotherapy, the patient’s condition worsened; however, a repeat biopsy identified prostate tuberculosis, and recovery was achieved after appropriate anti-tuberculosis therapy (23). Mário Henrique Magalhães Barros et al. and Hideki Asakawa et al. reported cases in which biopsy pathology initially indicated cervical lymph node and gastric tuberculosis. However, these patients failed to respond to anti-tuberculosis treatment and repeat biopsies later confirmed cervical lymph node and gastric lymphoma, respectively (32, 38). These findings highlight the potential for diagnostic confusion between lymphoma and tuberculosis, emphasizing the need for early biopsy, histopathological analysis, and immunohistochemistry to establish a definitive diagnosis. In cases where a patient’s condition fails to improve with initial treatment, repeat or multiple biopsies should be considered to rule out an alternative pathology and ensure timely and appropriate therapeutic intervention.

**Table 3:** Cases of misdiagnosis between lymphoma and tuberculosis have been reported in the English literature.

Source	Publication time	Age (years)/Sex	Symptoms	Misdiagnosis	Diagnosis	Treatment	Prognosis
Min Bai et al. (23)	2024	60/F	Loss of appetite	Prostate diffuse large B-cell lymphoma	Prostatic TB	R-CHOP chemotherapy was switched to anti-TB treatment	Alive
Nurafiqah Farhana Muhd Yazid et al. (24)	2024	38/M	Fever, general fatigue, confusion, cough, loss of appetite, and weight loss	TB of the central nervous system	Diffuse large B-cell lymphoma of the central nervous system	Anti-TB treatment	Died
Min Gu et al. (25)	2023	66/M	Cough, expectoration, dyspnea after activity, dizziness, tinnitus, night sweats, weight loss, and fatigue	Pulmonary TB	Pulmonary mucosa-associated lymphoid tissue lymphoma	Anti-CD20 monoclonal antibody (Rituximab)	Alive
Ying Wang et al. (26)	2022	43/M	Neck and facial swelling	TB of cervical lymph nodes	Diffuse large B-cell lymphoma of the cervical lymph nodes	R-DA-EPOCH chemotherapy	Alive
Henriette De La Garza et al. (27)	2021	31/F	Hoarseness and sore throat	Laryngeal natural killer (NK)/T-cell lymphoma	Laryngeal TB	Anti-TB treatment	Alive
Yaoyao Gong et al. (28)	2019	42/F	Melena and mild abdominal pain	Abdominal lymphoma.	Abdominal TB	Anti-TB treatment	Alive
Claudia Collu et al. (29)	2018	22/M	Thoracic pain, weight loss, latero-cervical, mediastinal, and abdominal lymphadenopathy	Pulmonary lymphoma	Pulmonary TB	Anti-TB treatment	Alive
Shasha Hou et al. (30)	2017	52/F	Irritable cough	Pulmonary lymphoma	Pulmonary TB	Anti-TB treatment	Alive
Qingxuan Wang et al. (31)	2016	21/M	Pain in the lower right abdomen	Lymphoma of the cervical lymph nodes	TB of cervical lymph nodes	Anti-TB treatment	Alive
Mário Henrique Magalhães Barros et al. (32)	2015	5/M	Enlarged left cervical region and evening fever	TB of cervical lymph nodes	Epstein-Barr virus-associated aggressive B-cell lymphoma of cervical lymph nodes	Anti-TB treatment was switched to chemotherapy and allogeneic bone marrow transplantation	Alive
Bahadır Öztürk et al. (33)	2015	23/F	Abdominal pain,vomiting,lost weight	Gastric lymphoma	Gastric TB	Anti-TB treatment	Alive
Piyush Ranjan et al. (34)	2015	21/M	Pain in the right inguinal region, intermittent fever, anorexia and weight loss	Extrapulmonary TB	Peripheral T-cell lymphoma of the duodenum	Anti-TB treatment	Died
Yew Li Dang et al. (35)	2014	58/F	Abdominal discomfort, weakness, headache, lethargy, confusion and gait difficult	TB of the central nervous system	Diffuse large B-cell lymphoma of the central nervous system	Anti-TB treatment	Died
Aleksandra Truszczyńska et al. (36)	2013	59/M	Girdle-like thoracolumbar pain, hyperthermia, weight loss, loss of appetite and leg weakness	Isolated vertebral lymphoma	Vertebral TB	Chemotherapeutic and radiotherapy treatments were switched to anti-TB treatment	Alive
Mehmet Buyukberber et al. (37)	2006	51/F	Fever, night sweats, abdominal swelling and pain	Peritoneal TB	Peritoneal non-Hodgkin lymphoma	Anti-TB treatment was switched to R-CHOP chemotherapy	Alive
Hideki Asakawa et al. (38)	2001	50/M	Epigastric discomfort	Gastric TB	Gastric T-cell lymphoma	Anti-TB treatment was switched to chemotherapy(cyclophosphamide, vincristine, and prednisone), a total gastrectomy was performed	Alive
P R Barker et al. (39)	1987	19/M	Generalized convulsion	Cerebral TB	Diffuse cerebral lymphoma of the histiocytic type	Anti-TB treatment	Died

TB, Tuberculosis; F, Female; M, Male; R-CHOP, Ruximab, cyclophosphamide, doxorubicin, vincristine, and prednisone; R-DA-EPOCH, Rituximab, dose-adjusted, etoposide, prednisone, oncovin, cyclophosphamide and hydroxydaunorubicin.

In this report, epididymal NK/T cell lymphoma and adrenal diffuse large B-cell lymphoma are specific subtypes of rare and challenging lymphomas. Both lymphoma and TB can involve multiple systems and organs, and their early clinical manifestations are not specific. However, the pathological diagnosis of lymphoma is affected by many factors, such as the location, staining, quality, duration of disease, and the experience of pathologists. When the pathology is inconsistent with the clinical diagnosis, multiple biopsies at different sites are more helpful to confirm the diagnosis. At the same time, in areas with a high incidence of TB, a positive TB immunological test is more common, and imaging examination may confuse lymphoma with TB. Therefore, in diagnosing adrenal lymphoma or tuberculosis, clinicians can not only make a diagnosis by biochemical and imaging examination results but also need to comprehensively analyze the patient’s symptoms, clinical signs, bacteriology, histopathology, and treatment efficacy.

## Data Availability

The original contributions presented in the study are included in the article material, further inquiries can be directed to the corresponding author.

## References

[1] Khoury JD Solary E Abla O Akkari Y Alaggio R Apperley JF. The 5th edition of the world health organization classification of haematolymphoid tumours: myeloid and histiocytic/dendritic neoplasms. Leukemia. (2022) 36:1703–19. doi: 10.1038/s41375-022-01613-1 PMC9252913 35732831

[2] Jiménez-Pérez JC Yoon MK. Natural killer T-cell lymphoma of the orbit: an evidence-based approach. Semin Ophthalmol. (2017) 32:116–24. doi: 10.1080/08820538.2016.1228405 27740876

[3] Nejjari H Ait Zine I Ammouri W Bernousssi Z Kabbaj H. Primary testicular extranodal NK/T-cell lymphoma nasal type associated with epstein-barr virus infection: A moroccan case report. Cureus. (2024) 16:e63361. doi: 10.7759/cureus.63361 39070361 PMC11283753

[4] Miranda-Filho A Piñeros M Znaor A Marcos-Gragera R Steliarova-Foucher E Bray F. Global patterns and trends in the incidence of non-Hodgkin lymphoma. Cancer Causes Control. (2019) 30:489–99. doi: 10.1007/s10552-019-01155-5 30895415

[5] Laurent C Casasnovas O Martin L Chauchet A Ghesquieres H Aussedat G. Adrenal lymphoma: presentation, management and prognosis. QJM. (2017) 110:103–9. doi: 10.1093/qjmed/hcw174 27795295

[6] Chakaya J Khan M Ntoumi F Aklillu E Fatima R Mwaba P. Global Tuberculosis Report 2020 - Reflections on the Global TB burden, treatment and prevention efforts. Int J Infect Dis. (2021) 113 Suppl 1:S7–S12. doi: 10.1016/j.ijid.2021.02.107 33716195 PMC8433257

[7] Rodriguez-Takeuchi SY Renjifo ME Medina FJ. Extrapulmonary tuberculosis: pathophysiology and imaging findings. Radiographics. (2019) 39:2023–37. doi: 10.1148/rg.2019190109 31697616

[8] Sun J Li G Zhang N Li S Chen R. Analysis of lymphoma presenting with pulmonary symptoms: report of 79 cases. Zhonghua Jie He He Hu Xi Za Zhi. (2014) 37(8):597–600. Chinese. 25351267

[9] Tse E Zhao WL Xiong J Kwong YL. How we treat NK/T-cell lymphomas. J Hematol Oncol. (2022) 15:74. doi: 10.1186/s13045-022-01293-5 35659326 PMC9164389

[10] Reneau JC ShIndiapina P Braunstein Z Youssef Y Ruiz M Farid S. Extranodal natural killer/T-cell lymphomas: current approaches and future directions. J Clin Med. (2022) 11:2699. doi: 10.3390/jcm11102699 35628826 PMC9145443

[11] Lim SH Hong JY Lim ST Hong H Arnoud J Zhao W. Beyond first-line non-anthracycline-based chemotherapy for extranodal NK/T-cell lymphoma: clinical outcome and current perspectives on salvage therapy for patients after first relapse and progression of disease. Ann Oncol. (2017) 28:2199–205. doi: 10.1093/annonc/mdx316 28911074

[12] Bi XW Wang H Zhang WW Wang JH Liu WJ Xia ZJ. PD-L1 is upregulated by EBV-driven LMP1 through NF-κB pathway and correlates with poor prognosis in natural killer/T-cell lymphoma. J Hematol Oncol. (2016) 9:109. doi: 10.1186/s13045-016-0341-7 27737703 PMC5064887

[13] Asano N Kato S Nakamura S. Epstein-Barr virus-associated natural killer/T-cell lymphomas. Best Pract Res Clin Haematol. (2013) 26:15–21. doi: 10.1016/j.beha.2013.04.002 23768637

[14] Asano N Suzuki R Kagami Y Ishida F Kitamura K Fukutani H. Clinicopathologic and prognostic significance of cytotoxic molecule expression in nodal peripheral T-cell lymphoma, unspecified. Am J Surg Pathol. (2005) 29:1284–93. doi: 10.1097/01.pas.0000173238.17331.6b 16160469

[15] Fox CP Civallero M Ko YH Manni M Skrypets T Pileri S. Survival outcomes of patients with extranodal natural-killer T-cell lymphoma: a prospective cohort study from the international T-cell Project. Lancet Haematol. (2020) 7:e284–94. doi: 10.1016/S2352-3026(19)30283-2 32105608

[16] Du W Yang JY Huang M Li JC Song F. A case report of epididymal testicular NK/T-cell lymphoma. J Pract Oncology. (2012) 27:664–5. doi: 10.13267/j.cnki.syzlzz.2012.06.011

[17] Tessler FN Tublin ME Rifkin MD. Ultrasound assessment of testicular and paratesticular masses. J Clin Ultrasound. (1996) 24:423–36. doi: 10.1002/(SICI)1097-0096(199610)24:8<423::AID-JCU3>3.0.CO;2-M 8884520

[18] Li S Wang Z Wu Z Zhuang H Xu Y. Clinical characteristics and outcomes of primary adrenal diffuse large B cell lymphoma in a large contemporary cohort: a SEER-based analysis. Ann Hematol. (2019) 98:2111–9. doi: 10.1007/s00277-019-03740-9 31227873

[19] Wang Y Ren Y Ma L Li J Zhu Y Zhao L. Clinical features of 50 patients with primary adrenal lymphoma. Front Endocrinol (Lausanne). (2020) 11:595. doi: 10.3389/fendo.2020.00595 33071959 PMC7541938

[20] Hans CP Weisenburger DD Greiner TC Gascoyne RD Delabie J Ott G. Confirmation of the molecular classification of diffuse large B-cell lymphoma by immunohistochemistry using a tissue microarray. Blood. (2004) 103:275–82. doi: 10.1182/blood-2003-05-1545 14504078

[21] Lu TX Miao Y Wu JZ Gong QX Liang JH Wang Z. The distinct clinical features and prognosis of the CD10^+^MUM1^+^ and CD10^−^Bcl6^−^MUM1^−^ diffuse large B-cell lymphoma. Sci Rep. (2016) 6:20465. doi: 10.1038/srep20465 26857366 PMC4746587

[22] Jiang LM Peng JX He YF Hu ZW Wang LJ Li XL. Report of one case with primary adrenal lymphoma misdiagnosed as adrenal tuberculosis [chinese. Chin J Endocrinol Metab. (2015) 31:541–1. doi: 10.3760/cma.j.issn.1000-6699.2015.06.017

[23] Bai M Yu Q Yuan L Zhao Y Zheng M Su L. Prostate tuberculosis mimicking Malignancy on 18F-FDG PET/CT in a patient with diffuse large B-cell lymphoma: A case report. Med (Baltimore). (2024) 103:e38296. doi: 10.1097/MD.0000000000038296 PMC11142781 39259096

[24] Muhd Yazid NF Che Ros MIA Setia SA. A rare case of primary CNS lymphoma in an HIV-positive patient mimicking CNS tuberculosis. Cureus. (2024) 16:e62426. doi: 10.7759/cureus.62426 39011202 PMC11248490

[25] Gu M Ji D Lu Y Ping G Yan C. Rare primary pulmonary mucosa-associated lymphoid tissue lymphoma misdiagnosed with tuberculosis: A case report. Med (Baltimore). (2023) 102:e36125. doi: 10.1097/MD.0000000000036125 PMC10659730 37986314

[26] Wang Y Chen M Ni C Tong J Chen P Zhang Y. Case report: primary mediastinal large B-cell lymphoma invasion of extranodal thyroid tissue mimicking tuberculosis and confounded by similar ultrasonic appearance. Front Oncol. (2022) 12:879295. doi: 10.3389/fonc.2022.879295 35664739 PMC9159155

[27] De La Garza H Flores R Grosu HB. A case of laryngeal tuberculosis mimicking lymphoma. Cureus. (2021) 13:e13744. doi: 10.7759/cureus.13744 33842122 PMC8022678

[28] Gong Y Li S Rong R Chen X Jiang L. Isolated gastric varices secondary to abdominal tuberculosis mimicking lymphoma: a case report. BMC Gastroenterol. (2019) 19:78. doi: 10.1186/s12876-019-0998-9 31138138 PMC6540571

[29] Collu C Fois A Crivelli P Tidore G Fozza C Sotgiu G. A case-report of a pulmonary tuberculosis with lymphadenopathy mimicking a lymphoma. Int J Infect Dis. (2018) 70:38–41. doi: 10.1016/j.ijid.2018.02.011 29477363

[30] Hou S Shen J Tan J. Case report: multiple systemic disseminated tuberculosis mimicking lymphoma on 18F-FDG PET/CT. Med (Baltimore). (2017) 96:e7248. doi: 10.1097/MD.0000000000007248 PMC5521880 28723740

[31] Wang Q Chen E Cai Y Zhang X Li Q Zhang X. A case report: systemic lymph node tuberculosis mimicking lymphoma on 18F-FDG PET/CT. Med (Baltimore). (2016) 95:e2912. doi: 10.1097/MD.0000000000002912 PMC4782873 26945389

[32] Barros MH Leite E Chabay P Morais V Stefanoff G Hassan R. Diagnosing lymphoma in a setting with a high burden of infection: a pediatric case of Epstein-Barr virus-associated aggressive B-cell lymphoma with t(8;14) (q23;q32) and extensive necrosis mimicking tuberculosis. Rev Soc Bras Med Trop. (2015) 48:108–11. doi: 10.1590/0037-8682-0153-2014 25860476

[33] Öztürk B Nural MS Ecemiş Ö Danacı M. Imaging findings of an isolated gastric tuberculosis case mimicking lymphoma and infiltrative gastric cancer. Turk J Gastroenterol. (2015) 26:65–6. doi: 10.5152/tjg.2015.5992 25698276

[34] Ranjan P Dutta S Kakkar A Goyal A Vikram NK Sharma MC. T-cell lymphoma masquerading as extrapulmonary tuberculosis: case report and review of literature. J Family Med Prim Care. (2015) 4:280–3. doi: 10.4103/2249-4863.154677 PMC4408718 25949984

[35] Dang YL Hor JY Chia YK Lim TT Eow GB. Suprasellar lymphoma masquerading as tuberculosis of the central nervous system. Acta Neurol Belg. (2014) 114:239–41. doi: 10.1007/s13760-013-0217-3 23757110

[36] Truszczyńska A Nowak-Misiak M Rąpała K Walczak P. Tuberculosis of the spine masquerading as a spine lymphoma. A case report and discussion of diagnostic and therapeutic traps. Neurol Neurochir Pol. (2013) 47:189–93. doi: 10.5114/ninp.2013.33823 23650010

[37] Buyukberber M Sevinc A Cagliyan CE Gulsen MT Sari I Camci C. Non-Hodgkin lymphoma with high adenosine deaminase levels mimicking peritoneal tuberculosis: an unusual presentation. Leuk Lymphoma. (2006) 47:565–8. doi: 10.1080/10428190500395472 16396783

[38] Asakawa H Tsuji M Tokumine Y Kashihara T Okuno M Takenaka R. Gastric T-cell lymphoma presenting with epithelioid granulomas mimicking tuberculosis in regional lymph nodes. J Gastroenterol. (2001) 36:190–4. doi: 10.1007/s005350170128 11291883

[39] Barker PR Kwan JS Patten JP. Primary cerebral lymphoma mimicking tuberculosis: case report. J Neurol Neurosurg Psychiatry. (1987) 50:358–60. doi: 10.1136/jnnp.50.3.358 PMC1031807 3559618

